# Independent and joint associations of fatty liver index and physical activity with mortality in adults with hypertension: a nationwide cohort study

**DOI:** 10.1038/s41440-026-02600-0

**Published:** 2026-04-07

**Authors:** Yunmin Han, Younghwan Choi, Yeon Soo Kim

**Affiliations:** 1 Department of Physical Education, Seoul National University, Seoul, South Korea; 2 Institute of Sports Science, Seoul National University, Seoul, South Korea

**Keywords:** Hypertension, Nonalcoholic fatty liver disease, Physical activity, Cardiovascular disease, Digital hypertension.

## Abstract

Hypertension and non-alcoholic fatty liver disease (NAFLD) frequently coexist and share metabolic pathways that elevate cardiovascular and all-cause mortality risk. Although physical activity (PA) is known to reduce cardiovascular risk, its impact among individuals with both hypertension and NAFLD remains unclear. This nationwide cohort study examined the independent and joint associations of PA and fatty liver burden with mortality in adults with hypertension. We analyzed 139,015 individuals aged ≥ 20 years who participated in the Korean National Health Insurance Service health screening program between 2009 and 2012 and were followed through 2021. Fatty liver burden was assessed using the Fatty Liver Index (FLI) and categorized as < 30, 30–59, or ≥ 60. PA levels were self-reported and classified as < 500, 500–999, and ≥ 1000 MET-min/week. Over a median follow-up of 9.1 years, 12,281 deaths occurred, including 2013 from cardiovascular causes. Higher FLI (≥ 60) was associated with significantly increased all-cause (HR 1.35, 95% CI 1.26–1.44) and cardiovascular mortality (HR 1.32, 95% CI 1.12–1.56). In contrast, higher PA (≥ 1000 MET-min/week) was consistently associated with lower mortality across all FLI categories, with the strongest benefit among those with FLI < 30 (HR 0.51 for all-cause mortality and 0.49 for CVD mortality). Importantly, high PA levels remained protective even in individuals with severe fatty liver burden. These findings suggest that regular PA substantially reduces mortality risk in adults with hypertension, regardless of underlying fatty liver severity. PA promotion should be considered an essential strategy in managing hypertension and related metabolic dysfunction.

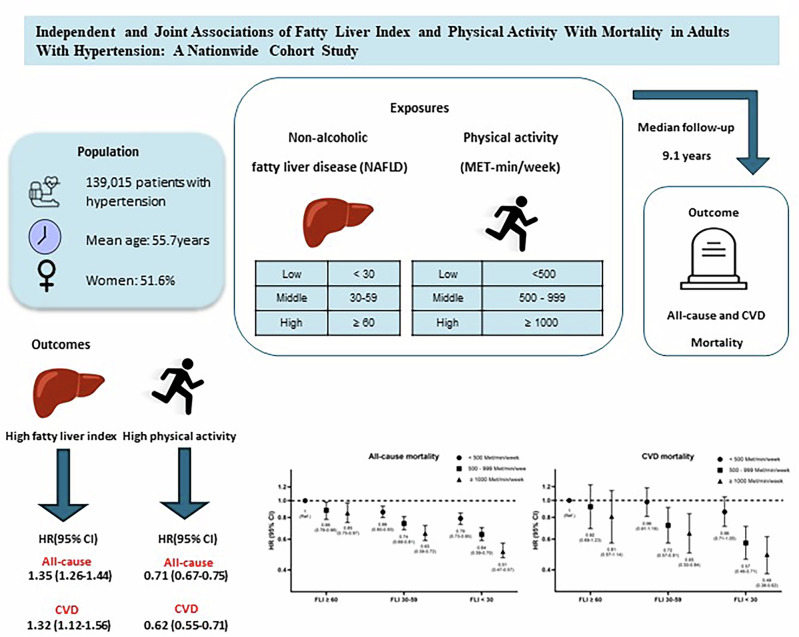

## Introduction

Hypertension is among the most prevalent chronic conditions worldwide and a leading risk factor for cardiovascular disease (CVD) and all-cause mortality [1]. The global burden of hypertension is further compounded by its frequent coexistence with non-alcoholic fatty liver disease (NAFLD), a significant contributor to adverse cardiovascular outcomes [2]. NAFLD is increasingly recognized as part of the global burden of CVD because of its direct and indirect effects on cardiovascular health [3]. Furthermore, hypertension and NAFLD are believed to share a bidirectional association, with each condition potentially influencing the onset or progression of the other through shared metabolic and inflammatory mechanisms [4]. In patients with hypertension, the high prevalence of NAFLD increases the risk of mortality and morbidity, as these conditions often interact synergistically [5]. Understanding the combined impact of hypertension and NAFLD is essential for developing effective strategies to mitigate cardiovascular risk in affected populations.

Physical activity (PA) is a well-established protective factor against CVD and metabolic disorders [6]. Regular PA regulates blood pressure, reduces hepatic fat accumulation, and improves insulin sensitivity, potentially mitigating NAFLD [7]. Additionally, PA is important in maintaining vascular health, reducing inflammation, and enhancing cardiovascular function, which contribute to lowering the risk of CVD-related and all-cause mortality in diverse populations [6, 8]. However, the interaction of PA and NAFLD that could influence mortality outcomes, particularly in individuals with hypertension, is unclear.

Currently, research has predominantly focused on the independent effects of NAFLD and PA on mortality with limited exploration of their combined effects, particularly in hypertensive populations. Furthermore, previous studies have often overlooked the potential interplay between PA and NAFLD in modulating CVD and all-cause mortality risk. Understanding these relationships is critical for developing effective targeted strategies to reduce the mortality risk in patients with hypertension and comorbid NAFLD.

This study aimed to examine the independent associations of NAFLD and PA with all-cause and CVD mortality and analyze dose–response relationships. Additionally, this study evaluated the joint association between NAFLD and PA in influencing mortality outcomes of individuals with hypertension.

Point of view

- Clinical relevance: Regular PA markedly reduces all-cause and cardiovascular mortality in adults with hypertension, even in the presence of severe fatty liver burden.
- Future direction: Prospective intervention studies are needed to define optimal PA prescriptions for adults with hypertension and non-alcoholic fatty liver disease.
- Consideration for the Asian population: Given the high metabolic risk at lower adiposity in Asian populations, early and sustained promotion of PA is particularly critical.

## Methods

### Data sources and study population

This study used data from the National Health Insurance Service-National Sample Cohort (NHIS-NSC), a pre-established, population-based cohort constructed by the National Health Insurance Service (NHIS) of South Korea. The NHIS-NSC provides anonymized, nationwide healthcare data, including demographic information, medical diagnoses coded according to the International Classification of Diseases, 10th Revision (ICD-10), treatment records, prescription details, and health examination data. The database contains information on lifestyle factors, such as smoking, alcohol consumption, and PA, collected during biennial health checkups [9]. All diagnoses were recorded in the NHIS database using ICD-10 codes.

The study population comprised adults aged ≥ 20 years who participated in general health screenings conducted from 2009 to 2012. Individuals were defined as having hypertension if they met any of the following criteria at baseline: (1) an ICD-10 diagnosis of hypertension (I10–I13, I15); (2) systolic blood pressure ≥140 mmHg or diastolic blood pressure ≥90 mmHg measured during the health screening; or (3) a prescription record for antihypertensive medication before the first available health screening record, defined as the earliest biennial screening data available in the NHIS database for each participant [10].

**Fig. 1:**
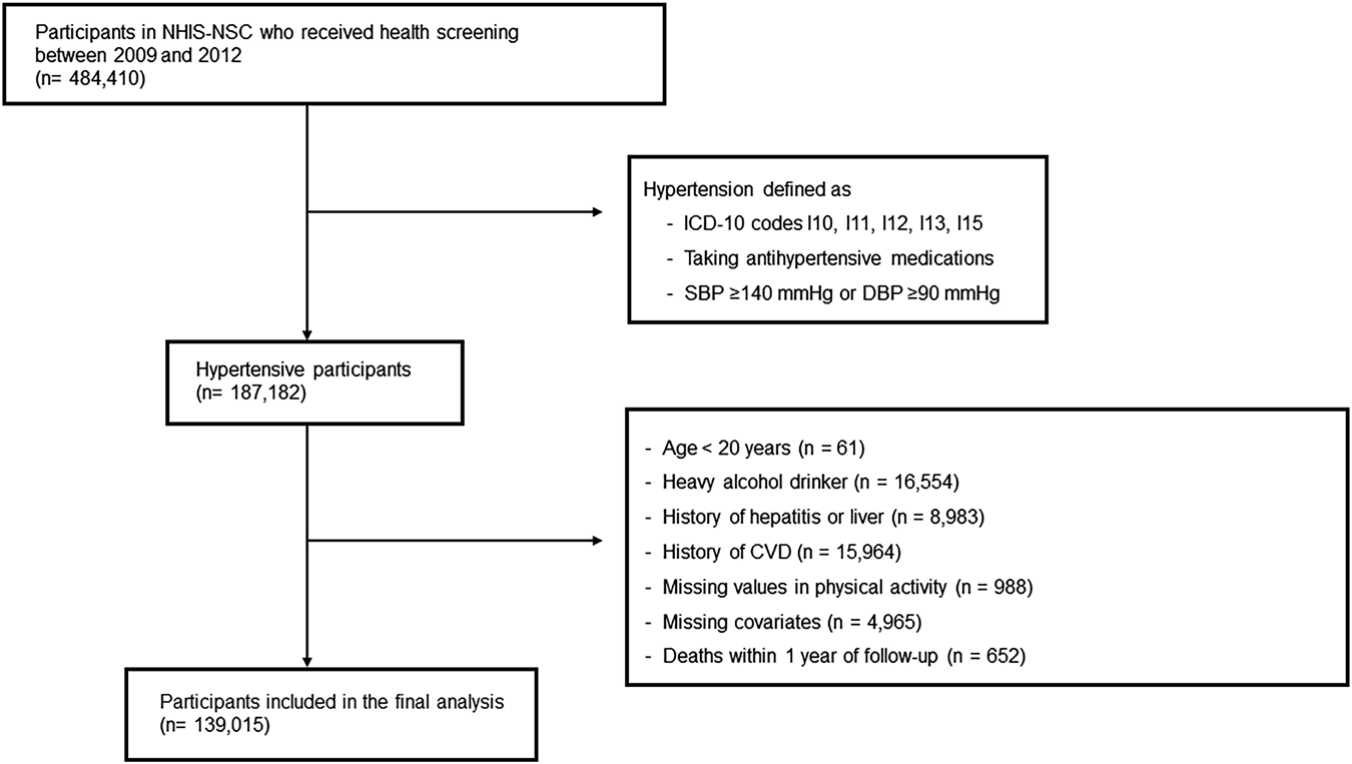
Flowchart for study participants

The exclusion criteria were as follows; individuals aged < 20 years, heavy alcohol drinkers (alcohol consumption ≥30 g/day for men and ≥20 g/day for women), participants with a history of CVD, including myocardial infarction (ICD-10 codes I21, I22) and ischemic stroke (ICD-10 codes I63, I64), participants with a history of liver disease, including liver cirrhosis (ICD-10 codes K70, K74) and hepatitis (ICD-10 codes B15–B19), individuals with missing data on PA variables or key covariates, and those who died within one year of their baseline health examination. Therefore, the final analysis included 139,015 participants (Fig. 1). This study protocol was reviewed and approved by the Institutional Review Board of Seoul National University (IRB No. E2408/004-014). This study utilized de-identified data from the Korean National Health Insurance Service (NHIS; approval number NHIS-2024-10-2-01), and the requirement for informed consent was waived in accordance with institutional guidelines.

### Exposures

Leisure-time PA level was assessed using self-reported structured questionnaires based on a seven-day recall method. Participants reported the number of days per week they engaged in moderate-to-vigorous physical activity (MVPA). Regular exercise was defined as engaging in moderate-intensity PA for ≥ 30 min on ≥ 5 days per week or vigorous-intensity PA for ≥ 20 min on ≥ 3 days per week.

PA intensity levels were categorized using MET values: 3.0 METs for light PA, 4.0 METs for moderate PA, and 8.0 METs for vigorous PA. PA-related energy expenditure was calculated in MET-minutes per week by multiplying the MET value for each activity by its frequency and duration, then summing the total MET-min from all MVPA. Based on weekly MET-min levels, PA was classified into three categories: < 500, 500–999, and ≥ 1000 MET-min/week [11–13].

NAFLD was defined using the fatty liver index (FLI), a validated surrogate marker of hepatic steatosis [14]. The FLI was by combining clinical and biochemical parameters, including triglyceride (TG) levels, body mass index (BMI), waist circumference (WC), and gamma-glutamyl transferase (GGT) levels. The FLI is calculated as follows:

$${FLI}=\frac{{e}^{0.953\cdot {{\mathrm{ln}}}\left({{{\rm{TG}}}}\right)+0.139\cdot {{{\rm{BMI}}}}+0.718\cdot {{\mathrm{ln}}}\left({{{\rm{GGT}}}}\right)+0.053\cdot {{{\rm{WC}}}}-15.745}}{1+{e}^{0.953\cdot {{\mathrm{ln}}}\left({{{\rm{TG}}}}\right)+0.139\cdot {{{\rm{BMI}}}}+0.718\cdot {{\mathrm{ln}}}\left({{{\rm{GGT}}}}\right)+0.053\cdot {{{\rm{WC}}}}-15.745}}\cdot 100\,$$

Participants were classified into three groups based on their FLI scores; ≥ 60 (with NAFLD), 30–59 (intermediate probability of fatty liver), and < 30 (without NAFLD) [15, 16].

### Outcomes

The primary outcomes were all-cause and CVD mortality rates. Mortality data were obtained from the Korean NHIS database linked to the National Death Registry. All-cause mortality was defined as death from any cause, and CVD mortality was classified as the underlying cause of death based on ICD-10 codes I00–I99. Follow-up was conducted until the date of death, loss to follow-up, or December 31, 2019, whichever came first.

### Covariates

Covariates included lifestyle factors, socioeconomic status, anthropometric measures, and chronic disease diagnoses, which were derived from self-reported questionnaires and clinical records from the Korean NHIS database. Age was categorized into three groups: 20–39, 40–59, and ≥ 60 years. Smoking status was categorized as never smoker, ex-smoker, and current smoker, whereas alcohol consumption (g/day) was calculated by multiplying the amount of alcohol consumed per occasion by the frequency of alcohol intake weekly. Income level was assessed based on health insurance premium deciles, with participants classified into the lowest 20% or remaining 80%. BMI was classified into three categories: normal weight, overweight, and obesity. Diabetes mellitus was defined as a diagnosis recorded under ICD-10 codes E10–E14 or receiving treatment for diabetes. Dyslipidemia was identified using the ICD-10 code E78 or a record of treatment for dyslipidemia. Antihypertensive medication use was defined as having at least one prescription record for antihypertensive agents during the baseline assessment period. Baseline systolic blood pressure (SBP) was categorized as < 140 mmHg and ≥ 140 mmHg based on the health screening measurement.

### Statistical analysis

Continuous variables were compared across groups using analysis of variance (ANOVA) and presented as means ± standard deviation (SD). Categorical variables were summarized as counts and percentages, and differences between groups were assessed using the Chi-square test to identify significant associations.

To investigate the independent and joint associations of FLI and PA with all-cause and CVD mortality, multivariate Cox proportional hazards models were employed. Three models were constructed to account for potential confounding variables. Model 1 is adjusted for age and sex. Model 2 included additional adjustments for current smoking status, alcohol consumption, and income level. Model 3a was further adjusted for diabetes, dyslipidemia, baseline SBP categories, and antihypertensive medication use, with additional mutual adjustment for PA level and FLI categories depending on the main exposure of interest. Model 3b additionally included BMI categories. A restricted cubic spline model was used to evaluate the dose–response relationships between PA, FLI, and mortality outcomes based on the distribution of PA and FLI to assess the potential curvilinearity in these associations. Given potential biological differences between sexes, all analyses were additionally stratified by sex. Sex-specific Cox proportional hazards models were fitted using the same covariate structure as the main analysis. In addition, we examined the independent associations of individual covariates with all-cause and CVD mortality using fully adjusted Cox proportional hazards models. Hazard ratios (HRs) and 95% confidence intervals (CIs) for each covariate were estimated from the fully adjusted model that included age, sex, smoking status, alcohol consumption, income level, BMI categories, diabetes, dyslipidemia, baseline SBP categories, and antihypertensive medication use.

Joint analyses were performed to examine the combined effects of PA and FLI on mortality by categorizing the participants into nine groups. These groups were defined based on PA levels (< 500, 500–999, and ≥ 1000 MET-min/week) and FLI categories (< 30, 30–59, and ≥ 60). Fully adjusted models were used to analyze these categories to understand the influence of varying levels of PA and FLI on mortality outcomes. To evaluate the joint association of PA and FLI, we tested for multiplicative interaction by comparing models with and without an interaction term using the log-likelihood ratio test.

To test the robustness of the findings, sensitivity analyses were conducted by excluding participants who died within the first three years of follow-up to reduce potential bias from reverse causation. The proportional hazards assumption of the Cox models was graphically evaluated using log-minus-log plots. All statistical analyses were performed using R software version 4.3.0. Statistical significance was set at *p* < 0.05.

## Results

### Baseline characteristics

A total of 139,015 participants (mean age; 55.7 ± 13.1 years, 51.6% women) were included, with a median follow-up of 9.1 years. Among these, 12,281 deaths occurred, including 2013 from CVD. Participants with higher PA levels had a higher prevalence of diabetes (26.9%) and dyslipidemia (43.8%). Contrastingly, those with high NAFLD (≥60 FLI) had higher BMI, waist circumference, and worse cardiometabolic markers, including diabetes (33.8%) and dyslipidemia (51.3%). The detailed baseline characteristics of the participants are presented in Table 1.

**Table 1:** Baseline characteristics by physical activity and fatty liver index

		PA categories			FLI categories		
	Overall	< 500 MET-min/week	500–999 MET-min/week	≥ 1000 MET-min/week	< 30	30–59	≥ 60
	(*n* = 139,015)	(*n* = 76,862)	(*n* = 36,948)	(*n* = 25,205)	(*n* = 73,532)	(*n* = 41,412)	(*n* = 24,071)
Women (*n*, %)	71,777 (51.6)	42,799 (55.7)	17,792 (48.2)	11,186 (44.4)	47,640 (64.8)	17,693 (42.7)	6444 (26.8)
Age (years)	55.7 ± 13.1	56.0 ± 13.4	55.1 ± 13.1	55.8 ± 12.1	56.3 ± 13.4	56.8 ± 12.4	52.0 ± 12.6
Height (cm)	161.3 ± 9.5	160.5 ± 9.6	162.1 ± 9.3	162.7 ± 9.0	159.3 ± 8.9	162.3 ± 9.5	165.8 ± 9.3
Weight (kg)	64.6 ± 11.9	63.9 ± 12.0	65.1 ± 12.0	65.8 ± 11.5	58.1 ± 8.5	68.0 ± 8.8	78.3 ± 11.6
Waist circumference (cm)	83.1 ± 8.9	83.0 ± 9.1	83.1 ± 8.8	83.2 ± 8.5	77.7 ± 6.7	86.6 ± 5.5	93.4 ± 7.2
Body mass index (kg/m^2^)	24.7 ± 3.3	24.7 ± 3.4	24.7 ± 3.3	24.7 ± 3.1	22.8 ± 2.4	25.8 ± 2.3	28.4 ± 3.3
Systolic blood pressure (mmHg)	131.6 ± 16.2	131.6 ± 16.5	131.8 ± 16.1	131.5 ± 15.7	129.2 ± 16.6	133.3 ± 15.4	136.1 ± 15.3
Diastolic blood pressure (mmHg)	81.8 ± 11.1	81.7 ± 11.2	82.0 ± 11.1	81.6 ± 10.9	80.0 ± 11.1	82.8 ± 10.7	85.5 ± 10.9
Fasting glucose (mg/dL)	102.7 ± 27.3	102.6 ± 27.8	102.6 ± 26.6	103.3 ± 26.6	98.4 ± 22.8	105.4 ± 28.8	111.3 ± 33.7
Total cholesterol (mg/dL)	199.3 ± 40.6	200.0 ± 40.4	199.2 ± 42.1	197.6 ± 39.1	194.1 ± 39.0	202.7 ± 42.0	209.6 ± 40.5
HDL-C (mg/dL)	54.5 ± 28.4	54.5 ± 27.6	54.5 ± 33.5	54.6 ± 22.1	57.1 ± 27.2	51.9 ± 24.9	51.0 ± 36.0
LDL-C (mg/dL)	118.0 ± 61.0	118.7 ± 68.2	117.5 ± 51.4	116.7 ± 49.5	117.5 ± 54.1	120.1 ± 53.4	116.1 ± 87.3
Triglycerides (mg/dL)	146.2 ± 105.5	148.1 ± 105.3	146.3 ± 104.5	140.4 ± 107.5	103.5 ± 48.2	163.8 ± 75.1	246.5 ± 177.0
AST (U/L)	26.5 ± 18.3	26.5 ± 19.3	26.5 ± 17.1	26.7 ± 17.0	23.7 ± 12.5	26.5 ± 18.3	34.4 ± 29.8
ALT (U/L)	26.7 ± 24.1	26.8 ± 24.5	26.8 ± 23.9	26.4 ± 23.1	20.4 ± 15.6	28.7 ± 21.6	42.6 ± 37.7
GGT (U/L)	38.2 ± 49.0	37.9 ± 50.2	38.7 ± 47.4	38.1 ± 47.7	22.2 ± 15.8	40.8 ± 36.2	82.4 ± 90.0
Current smoker (%)	25,684 (18.5)	13,916 (18.1)	7,379 (20.0)	4,389 (17.4)	9,324 (12.6)	8,579 (20.7)	7,871 (32.7)
Alcohol drinking (g/day)	4.0 ± 7.0	3.6 ± 7.0	4.5 ± 7.2	4.7 ± 7.4	2.6 ± 5.5	4.7 ± 7.4	7.4 ± 8.7
Low-income level (%)	21,231 (15.3)	12,068 (15.7)	5563 (15.1)	3600 (14.3)	11,861 (16.1)	5981 (14.4)	3389 (14.1)
Diabetes (%)	35,000 (25.2)	19,051 (24.8)	9176 (24.8)	6773 (26.9)	14,885 (20.2)	11,981 (28.9)	8134 (33.8)
Dyslipidemia (%)	58,645 (42.2)	32,293 (42.0)	15,322 (41.5)	11,030 (43.8)	27,066 (36.8)	22,185 (53.6)	12,352 (51.3)

Data are presented as mean (standard deviation), or percentages

*PA* Physical activity, *FLI* Fatty liver index, *HDL-C* High-density lipoprotein cholesterol, *LDL-C* Low-density lipoprotein cholesterol

### All-cause and CVD mortality by FLI categories

The association between FLI and mortality is shown in Table 2. In model 1, participants with FLI of 30–59 had a lower risk of all-cause mortality (HR; 0.86, 95% CI; 0.82–0.91) than FLI < 30, whereas those with FLI ≥ 60 had a slightly increased risk (HR; 0.86, 95% CI; 0.82–0.91). After full adjustment (model 3), FLI ≥ 60 was significantly associated with higher risks of all-cause (HR; 1.33, 95% CI; 1.26–1.44) and CVD mortality (HR; 1.32, 95% CI; 1.12–1.56), with a significant trend across FLI (*p* < 0.001).

**Table 2:** Hazard ratios of all-cause and CVD mortality according to fatty liver index categories or physical activity levels

All-cause mortality	*N*	Events (*n*)			HR (95% CI)		
FLI categories			Model 1	Model 2		Model 3^a^	Model 3^b^
< 30	73,532	6837	1.00 (Reference)	1.00 (Reference)		1.00 (Reference)	1.00 (Reference)
30–59	41,412	3628	**0.81 (0.78─0.95)**	**0.82 (0.79─0.86)**		**0.81 (0.78─0.84)**	**1.13 (1.08─1.19)**
≥ 60	24,071	1816	**0.86 (0.82─0.91)**	**0.88 (0.84─0.93)**		**0.85 (0.80─0.89)**	**1.35 (1.26─1.44)**
*p* for trend			**< 0.001**	**< 0.001**		**< 0.001**	**< 0.001**

	*N*	Events (*n*)			HR (95% CI)		
MVPA level			Model 1	Model 2		Model 3^a^	Model 3^b^
< 500 MET-min/week	76,862	7470	1.00 (Reference)	1.00 (Reference)		1.00 (Reference)	1.00 (Reference)
500–999 MET-min/week	36,948	2962	**0.83 (0.79─0.87)**	**0.84 (0.80─0.87)**		**0.83 (0.80─0.87)**	**0.84 (0.80─0.88)**
≥ 1000 MET-min/week	25,205	1849	**0.68 (0.64─0.71)**	**0.70 (0.66─0.73)**		**0.69 (0.66─0.73)**	**0.71 (0.67─0.75)**
*p* for trend			**< 0.001**	**< 0.001**		**< 0.001**	**< 0.001**

CVD mortality	*N*	Events (*n*)			HR (95% CI)		
FLI categories			Model 1	Model 2		Model 3^a^	Model 3^b^
< 30	73,532	1141	1.00 (Reference)	1.00 (Reference)		1.00 (Reference)	1.00 (Reference)
30–59	41,412	608	**0.85 (0.77─0.94)**	**0.86 (0.78─0.95)**		**0.84 (0.76─0.92)**	**1.19 (1.06─1.34)**
≥ 60	24,071	264	**0.84 (0.73─0.96)**	**0.85 (0.75─0.98)**		**0.80 (0.69─0.92)**	**1.32 (1.12─1.56)**
*p* for trend			**< 0.001**	**0.003**		**< 0.001**	**< 0.001**

	*N*	Events (*n*)			HR (95% CI)		
MVPA level			Model 1	Model 2		Model 3^a^	Model 3^b^
<500 MET-min/week	76,862	1297	1.00 (Reference)	1.00 (Reference)		1.00 (Reference)	1.00 (Reference)
500–999 MET-min/week	36,948	439	**0.70 (0.63─0.79)**	**0.71 (0.64─0.79)**		**0.78 (0.70─0.87)**	**0.72 (0.64─0.80)**
≥1000 MET-min/week	25,205	277	**0.59 (0.52─0.68)**	**0.61 (0.54─0.70)**		**0.74 (0.65─0.84)**	**0.62 (0.55─0.71)**
*p* for trend			**< 0.001**	**< 0.001**		**< 0.001**	**< 0.001**

Model 1: Adjusted for age and sex

Model 2: Adjusted for age, sex, smoking status, alcohol consumption, and income level

Model 3^a^: Adjusted for age, sex, smoking status, alcohol consumption, income level, diabetes, dyslipidemia, baseline SBP categories, antihypertensive medication use, and mutually adjusted for PA level and FLI categories, depending on the main exposure of interest

Model 3^b^: Model 3^a^ plus BMI categories

Bold values indicate statistical significance (*P* < 0.05)

### All-cause and CVD mortality by MVPA levels

Increased MVPA reduces the mortality risk. Participants with 500–999 MET-min/week showed lower all-cause mortality (HR; 0.84, 95% CI; 0.80–0.88), and those with ≥1000 MET-min/week had the lowest risk (HR; 0.71, 95% CI; 0.67–0.75; *p* < 0.001). Similar protective effects were observed for CVD mortality, with the highest MVPA group (≥1000 MET-min/week) showing the lowest risk (HR; 0.62, 95% CI; 0.55–0.71). In sex-stratified analyses, the inverse associations between MVPA and mortality were similar in men and women. However, the elevated CVD mortality risk associated with high FLI (≥60) was more pronounced in women than in men (Table 3).

**Table 3:** Hazard ratios of all-cause and CVD mortality according to fatty liver index categories or physical activity levels among men and women

All-cause mortality	Men			Women		
FLI categories	*N*	Events (*n*)	Fully adjusted HR (95% CI)	*N*	Events (*n*)	Fully adjusted HR (95% CI)
< 30	25,892	3353	1.00 (Reference)	47,640	3484	1.00 (Reference)
30–59	23,719	2172	**1.11 (1.04─1.18)**	17,693	1456	**1.16 (1.08─1.25)**
≥ 60	17,627	1241	**1.28 (1.18─1.39)**	6444	575	**1.47 (1.32─1.63)**
*p* for trend			**< 0.001**			**< 0.001**
MVPA level	*N*	Events (*n*)	Fully adjusted HR (95% CI)	*N*	Events (*n*)	Fully adjusted HR (95% CI)
< 500 MET-min/week	34,603	3697	1.00 (Reference)	42,799	3773	1.00 (Reference)
500–999 MET-min/week	19,516	1784	**0.89 (0.84─0.94)**	17,792	1178	**0.79 (0.74─0.84)**
≥ 1000 MET-min/week	14,019	1285	**0.78 (0.73─0.83)**	11,186	564	**0.62 (0.57─0.68)**
*p* for trend			**< 0.001**			**< 0.001**

CVD mortality	Men			Women		
FLI categories	*N*	Events (*n*)	Fully adjusted HR (95% CI)	*N*	Events (*n*)	Fully adjusted HR (95% CI)
< 30	25,892	461	1.00 (Reference)	47,640	680	1.00 (Reference)
30–59	23,719	354	**1.20 (1.02─1.41)**	17,693	254	1.15 (0.97─1.37)
≥ 60	17,627	161	1.09 (0.87─1.37)	6444	103	**1.61 (1.26─2.07)**
*p* for trend			**0.268**			**< 0.001**
MVPA level	*N*	Events (*n*)	Fully adjusted HR(95% CI)	*N*	Events (*n*)	Fully adjusted HR(95% CI)
< 500 MET-min/week	34,603	565	1.00 (Reference)	42,799	732	1.00 (Reference)
500–999 MET-min/week	19,516	225	**0.72 (0.61─0.84)**	17,792	214	**0.73 (0.63─0.85)**
≥ 1000 MET-min/week	14,019	186	**0.71 (0.60─0.84)**	11,186	91	**0.52 (0.41─0.64)**
*p* for trend			**< 0.001**			**< 0.001**

Full adjusted Model: Adjusted for age, sex, smoking status, alcohol consumption, income level, diabetes, dyslipidemia, baseline SBP categories, antihypertensive medication use, BMI categories, and mutually adjusted for PA level and FLI categories, depending on the main exposure of interest. Bold values indicate statistical significance (*P* < 0.05)

**Table 4:** Independent association of individual covariates with all-cause and CVD mortality

All-cause mortality			
Variable	Total *N*	Events (*n*)	Fully adjusted HR (95% CI)
Age			
20–39	15,255	141	1 (Reference)
40–59	67,240	1818	**3.24 (2.73─3.85)**
≥ 60	56,520	10,322	**19.72 (16.67─23.33)**
Sex			
Men	67,238	6766	1 (Reference)
Women	71,777	5515	**0.64 (0.61─0.67)**
Smoking status			
None	91,758	7797	1 (Reference)
Former smoker	21,663	2163	**1.11 (1.05─1.18)**
Current smoker	25,684	2411	**1.45 (1.37─1.53)**
Alcohol drinking	139,015	12,281	**0.98 (0.98-0.98)**
Income level			
Low	21,231	2039	1 (Reference)
High	117,784	10,242	1.06 (0.94─1.03)
BMI categories			
Normal	42,376	5135	1 (Reference)
Overweight	35,265	2994	**0.63 (0.69─0.66)**
Obesity	61,374	4152	**0.48 (0.45─0.50)**
Diabetes			
No	104,015	7244	1 (Reference)
Yes	35,000	5037	**1.46 (1.41─1.51)**
Dyslipidemia			
No	80,370	7311	1 (Reference)
Yes	58,645	4970	**0.78 (0.75─0.81)**
Baseline SBP categories			
SBP < 140	92,183	7960	1 (Reference)
SBP ≥ 140	46,832	4321	**1.06 (1.02─1.10)**
Antihypertensive medication			
No	81,589	5569	1 (Reference)
Yes	57,426	6712	**1.05 (1.01─1.09)**

CVD mortality			
Variable	Total *N*	Events (*n*)	Fully adjusted HR (95% CI)
Age			
20–39	15,555	26	1 (Reference)
40–59	67,240	254	**2.34 (1.56─3.51)**
≥ 60	56,520	1733	**18.05 (12.17─26.77)**
Sex			
Men	67,238	976	1 (Reference)
Women	71,777	1037	**0.79 (0.70─0.89)**
Smoking status			
None	91,668	1356	1 (Reference)
Former smoker	21,663	287	0.99 (0.85─1.15)
Current smoker	25,684	370	**1.50 (1.31─1.72)**
Alcohol drinking	139,015	2013	**0.98 (0.97-0.99)**
Income level			
Low	21,231	333	1 (Reference)
High	117,784	1680	0.97 (0.87─1.10)
BMI categories			
Normal	42,376	834	1 (Reference)
Overweight	35,265	526	**0.68 (0.60─0.76)**
Obesity	61,374	653	**0.46 (0.40─0.52)**
Diabetes			
No	104,015	1205	1 (Reference)
Yes	35,000	808	**1.42 (1.30─1.56)**
Dyslipidemia			
No	80,370	1174	1 (Reference)
Yes	58,645	839	**0.79 (0.72─0.86)**
Baseline SBP categories			
SBP < 140	92,183	1278	1 (Reference)
SBP ≥ 140	46,832	735	**1.14 (1.04─1.25)**
Antihypertensive medication			
No	81,589	879	1 (Reference)
Yes	57,426	1134	**1.15 (1.05─1.27)**

Fully adjusted model: Adjusted for age, sex, smoking status, alcohol consumption, income level, diabetes, dyslipidemia, baseline SBP categories, antihypertensive medication use, BMI categories, and mutually adjusted for PA level and FLI categories, depending on the main exposure of interest. Bold values indicate statistical significance (*P*< 0.05)

### Independent associations of covariates with mortality

The independent associations of covariates with all-cause and CVD mortality in the fully adjusted models are shown in Table 4. Older age was strongly associated with higher mortality (vs 20–39: 40–59 all-cause HR 3.24, 95% CI 2.73–3.85; CVD HR 2.34, 95% CI 1.56–3.51; ≥60 all-cause HR 19.72, 95% CI 16.67–23.33; CVD HR 18.05, 95% CI 12.17–26.77). Women had lower risk than men (all-cause HR 0.64, 95% CI 0.61–0.67; CVD HR 0.79, 95% CI 0.70–0.89), whereas current smoking and diabetes were associated with higher risk, and dyslipidemia with lower risk. Elevated SBP (≥140 mmHg) and antihypertensive medication use showed modestly higher risks for both outcomes.

### Dose–response relationships of PA and FLI with all-cause and CVD mortality

**Fig. 2:**
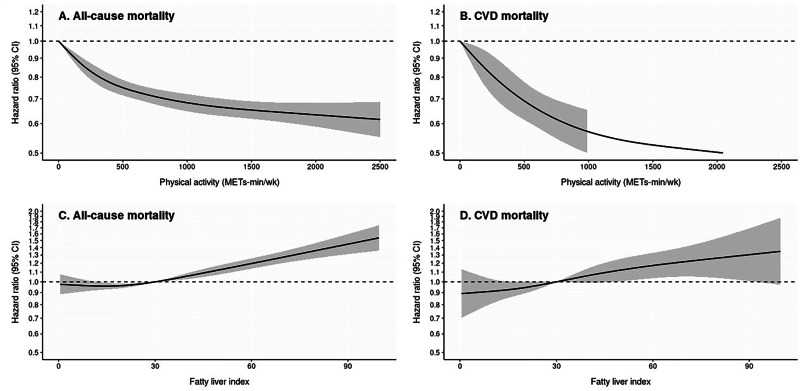
Dose–response relationship between PA, FLI, and all-cause and CVD mortality. **A** PA and all-cause mortality. **B** PA and CVD mortality. **C** FLI and all-cause mortality. **D** FLI and CVD mortality. The solid lines represent hazard ratios with 95% confidence intervals, estimated using restricted cubic splines with five knots. The fully adjusted model included age, sex, smoking status, alcohol consumption, income level, BMI categories, diabetes, dyslipidemia, baseline SBP categories, and antihypertensive medication use. PA and FLI were mutually adjusted when used as exposure variables. HR Hazard ratio, CI Confidence interval, CVD cardiovascular disease, PA physical activity, FLI fatty liver index, BMI Body mass index

The dose–response relationships among PA, FLI, and mortality risk are shown in Fig. 2. Increased PA levels (MET-min/week) were associated with reduced risks of all-cause (Fig. 2A) and CVD mortality (Fig. 2B), with the most pronounced decline observed < 1000 MET-min/week. Contrastingly, a high FLI, reflecting high NAFLD, was associated with an increased risk of all-cause (Fig. 2C) and CVD mortality (Fig. 2D). Mortality risks increased steadily at FLI values > 30 and sharply > 60, demonstrating a clear dose–response relationship between NAFLD severity and mortality.

### Joint associations between PA and FLI with all-cause and CVD mortality

**Fig. 3:**
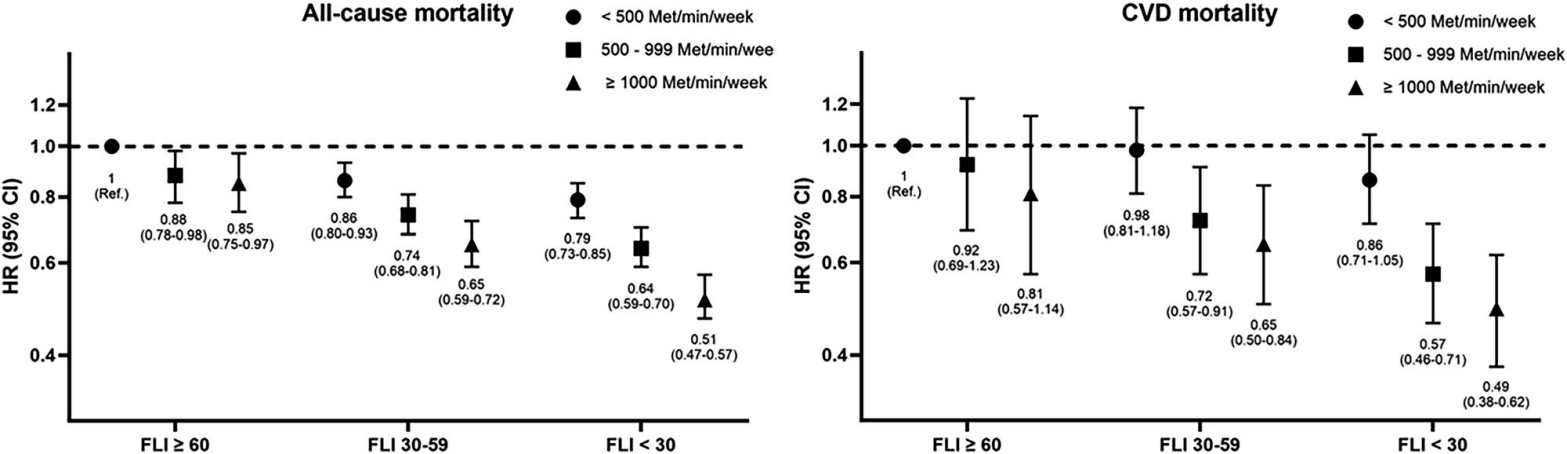
Joint association between PA, FLI, and all-cause and CVD mortality. The fully adjusted model included age, sex, smoking status, alcohol consumption, income level, BMI categories, diabetes, dyslipidemia, baseline SBP categories, and antihypertensive medication use. HR Hazard ratio, CI Confidence interval, CVD cardiovascular disease, PA physical activity, FLI fatty liver index, BMI Body mass index

Higher PA levels were consistently associated with a lower risk of all-cause and CVD mortality across all FLI categories (Fig. 3). The interaction between PA and FLI was statistically significant for all-cause mortality (*p* = 0.005) but not for cardiovascular mortality (*p* = 0.167). For all-cause mortality, participants with ≥ 1000 MET-min/week showed the greatest reduction in risk, with HRs of 0.85 (95% CI; 0.75–0.97), 0.65 (95% CI; 0.59–0.72), and 0.51 (95% CI; 0.47–0.57) in the FLI ≥ 60, 30–59, and < 30 group, respectively than those with < 500 MET-min/week. Similar trends were observed in CVD mortality. The HRs for participants with ≥ 1000 MET-min/week were 0.81 (95% CI; 0.57–1.14), 0.65 (95% CI; 0.50–0.84), and 0.49 (95% CI; 0.38–0.62) in the FLI ≥ 60, 30–59, and < 30 group, respectively.

### Sensitivity analysis

Excluding deaths within the first three years, similar trends were observed, confirming the robustness of the findings. Analyses of PA and FLI independently and jointly consistently demonstrated that higher PA and lower FLI severity reduced mortality risk, consistent with the main results (Supplementary Table 1).

## Discussion

This nationwide cohort study investigated the associations of fatty liver burden—assessed using the FLI—and PA with all-cause and cardiovascular mortality among patients with hypertension. Over a median follow-up of 9.1 years, higher levels of PA were consistently associated with lower risks of both all-cause and cardiovascular mortality across all FLI categories, including individuals with severe fatty liver burden. In contrast, higher FLI was progressively associated with increased mortality risk, highlighting that regular PA provides substantial protective effects even in hypertensive patients with advanced fatty liver.

Patients with hypertension are vulnerable to the progression of NAFLD and the associated risks of all-cause and cardiovascular mortality [17, 18]. Previous studies have shown that hypertension exacerbates the adverse metabolic effects of NAFLD, creating a synergistic impact on cardiovascular health [19, 20]. In our analysis, individuals with hypertension and high NAFLD (FLI ≥ 60) demonstrated significantly higher mortality risks than those without high NAFLD. Importantly, the protective effects of regular PA were observed across all NAFLD severity levels, suggesting that PA may be a critical intervention to mitigate these risks. Encouraging patients with hypertension to engage in MVPA, aiming for ≥ 500 MET-min/week, could attenuate NAFLD progression and improve overall and cardiovascular survival outcomes. These findings reinforce the need for integrated care approaches that combine NAFLD management with structured PA programs for the hypertensive population.

Our results align with and extend earlier research showing that NAFLD is associated with increased all-cause mortality through systemic inflammation and metabolic abnormalities [21, 22]. Although evidence of the impact of NAFLD on CVD mortality is inconsistent, previous Korean cohort studies have reported higher HRs for all-cause and cardiovascular mortality with increasing FLI scores [15, 23]. Our findings reinforce this relationship and highlight the heightened vulnerability of patients with hypertension, underscoring the need for comprehensive strategies to address both conditions simultaneously.

A notable finding of this study was the substantial change in the association between FLI and mortality after adjustment for BMI categories. While higher FLI appeared to be inversely associated with mortality in Model 3a, this association was reversed in Model 3b, revealing a clear dose–response relationship, with the highest FLI category associated with a 35% higher risk of all-cause mortality. This pattern may be partly explained by the obesity paradox frequently observed in Korean population-based cohorts, in which higher BMI is often associated with lower mortality [24]. In this context, BMI may function as a suppressor variable; its inclusion in the model attenuates the variance related to general adiposity, thereby revealing the adverse impact of hepatic fat accumulation. Our findings suggest that ectopic fat deposition in the liver represents a clinically distinct risk phenotype that cannot be adequately captured by BMI alone.

The protective effects of PA on all-cause and CVD mortality have been well-documented in previous studies [25]. PA confers physiological benefits by reducing mortality risk in various populations [26]. Particularly, MVPA is important in reducing the mortality associated with obesity and metabolic diseases [27, 28]. In a Korean cohort of individuals with hypertension, those who met the aerobic PA guidelines exhibited a 24% lower risk of all-cause and cardiovascular mortalities [29]. In comparison with those of the current study, our results suggest that PA may mitigate the increased mortality risks associated with NAFLD and hypertension. This study complements existing evidence by demonstrating that the protective effects of PA extend to individuals with hypertension and NAFLD, suggesting that PA may be a preventive measure and therapeutic intervention for managing mortality risks in this high-risk group. These findings emphasize the importance of promoting regular PA as part of comprehensive management strategies for patients with hypertension and NAFLD.

Previous studies have explored the interaction between NAFLD and PA, highlighting the protective role of PA in reducing the risk of NAFLD. For instance, individuals with objectively measured high PA levels were found to have a significantly lower risk of developing NAFLD (HR; 0.39, 95% CI; 0.21–0.70) than those with lower activity levels [30]. Another study reported a nonlinear inverse relationship between MVPA and incident NAFLD, with the risk decreasing sharply for every 100-min MVPA increment < 208 min (HR; 0.68, 95% CI; 0.57–0.81) and declining more moderately at higher activity levels (HR; 0.91, 95% CI; 0.84–0.99) [31]. Furthermore, reviews have shown that regular PA leads to significant reductions in intrahepatic fat and improvements in metabolic parameters in patients with NAFLD, suggesting its role as a cornerstone of disease management [32, 33].

Our study extended these findings by evaluating the interaction between FLI and PA in all-cause and cardiovascular mortality in individuals with hypertension. Unlike previous studies that focused on the general population or incident NAFLD, this study provides novel evidence that PA can attenuate the heightened mortality risk associated with NAFLD in adults with hypertension. By demonstrating that higher PA levels significantly reduced the risks of all-cause and CVD mortality, including patients with high NAFLD, our findings emphasize the importance of PA as a preventive strategy and therapeutic intervention to manage the combined burden of NAFLD and hypertension.

These findings can be explained by the underlying mechanisms linking NAFLD and PA with all-cause and cardiovascular mortality. NAFLD is associated with systemic inflammation, insulin resistance, dyslipidemia, and oxidative stress, which contribute to a heightened risk of cardiovascular events and mortality [34]. In patients with hypertension, these effects may be exacerbated by the synergistic effects of hypertension on vascular health and metabolic dysregulation [35, 36]. Conversely, PA has significant protective effects through multiple pathways, including the reduction of systemic inflammation, oxidative stress, and insulin resistance [37]. It helps lower the levels of pro-inflammatory markers, such as interleukin-6 and C-reactive protein, mitigates vasoconstriction by improving endothelial function and nitric oxide bioavailability, and enhances cardiovascular function by reducing arterial stiffness and blood pressure [38]. Additionally, PA decreases hepatic fat accumulation and improves lipid metabolism, further attenuating the metabolic stress and inflammation associated with NAFLD [39]. These combined effects contribute to improved metabolic and cardiovascular health, thereby reducing mortality risk in high-risk populations. Notably, our findings suggest that the beneficial effects of PA extend across all FLI levels owing to its capacity to counteract NAFLD-induced metabolic and cardiovascular risks. These results emphasize the critical role of regular PA, particularly at levels ≥ 500 MET-min/week, in mitigating adverse outcomes associated with FLI and hypertension. This highlights the importance of integrating PA interventions into the routine care of patients with hypertension and NAFLD to improve long-term health outcomes.

This study has several notable strengths, including the use of a large, nationally representative cohort with approximately 9 years of follow-up, which enables a robust evaluation of the long-term impact of NAFLD and PA on mortality in a hypertensive population. Notably, this is the first study to investigate the joint association of PA and NAFLD with all-cause and CVD mortality, specifically in individuals with hypertension, thereby providing novel insights into this high-risk population. However, this study had several limitations. First, the study relied on baseline measurements of the FLI and self-reported PA levels without accounting for potential changes over time. NAFLD severity and PA habits may have evolved during the follow-up period, and failure to account for such dynamic changes could limit the ability to capture longitudinal trends. Second, PA was assessed using self-reported questionnaires, which are subject not only to recall bias but also to social desirability bias, potentially leading to overestimation of PA levels. Future studies should consider objective tools, such as accelerometers or wearable trackers, to improve measurement accuracy. Third, although the FLI has been validated as a surrogate marker for hepatic steatosis and is widely used in population-based studies, it is not a gold-standard diagnostic tool. As such, it may not capture the full spectrum or severity of hepatic pathology. Future studies using imaging modalities (e.g., ultrasound, MRI) or histologic confirmation may provide more precise assessments of NAFLD. Fourth, although hypertension-related factors such as antihypertensive medication counts, medication adherence, and detailed blood pressure measures are clinically important, these variables were not sufficiently available in the dataset we accessed, which imposed practical limitations on their use in the analysis. Consequently, some residual confounding related to hypertension severity and treatment patterns may remain. Finally, the study population comprised only Koreans, which may limit the generalizability of the findings to other ethnic and cultural groups. Future research addressing these limitations, including repeated measures of FLI and PA, use of objective diagnostic tools, and studies in diverse populations, would enhance the validity and applicability of these findings.

### Perspective of Asia

Hypertension and NAFLD are highly prevalent in Asian populations, including Korea, where rapid epidemiological transition, increasing sedentary lifestyles, and metabolic disorders have contributed to a growing burden of cardiometabolic disease. Asian individuals tend to develop metabolic complications, including NAFLD and hypertension, at lower body mass indices compared with Western populations, underscoring the importance of early risk stratification and lifestyle-based prevention strategies. Using a nationwide Korean cohort with long-term follow-up, this study provides Asia-specific evidence that PA confers substantial survival benefits in hypertensive adults, even among those with a high fatty liver burden. These findings support the relevance of PA as a cornerstone intervention in Asian clinical practice and suggest that PA promotion may mitigate the heightened cardiometabolic vulnerability characteristic of Asian populations.

## Conclusion

Overall, higher levels of PA were associated with a reduced risk of all-cause and CVD mortality among adults with hypertension across all categories of fatty liver burden assessed by the FLI. These findings highlight the substantial protective role of regular PA in mitigating mortality risk in this high-risk population. Incorporating PA into the routine management of hypertension and metabolic dysfunction should be considered a key strategy to improve long-term health outcomes.

## Supplementary information

Supplementary Table 1

Supplementary Table 2

## Data Availability

The datasets used in this study are not publicly accessible, as they were derived from the National Health Insurance Service (NHIS) claims database. Additional data may be obtained from a third party with the necessary authorization, but they are not openly available. For access requests, please contact the National Health Insurance Sharing Service at https://nhiss.nhis.or.kr.
