# Supplementary material for: Independent and joint associations of fatty liver index and physical activity with mortality in adults with hypertension: a nationwide cohort study
Source: Hypertens Res. 2026 Apr 7;49(6):1839–52. doi: 10.1038/s41440-026-02600-0 (PMC13236582; doi:10.1038/s41440-026-02600-0)
Supplement: Supplementary file 1 — Supplementary Table 1 [file 41440_2026_2600_MOESM1_ESM.docx]

| **Supplementary Table 1. Hazard ratios of all-cause and CVD mortality according to fatty liver index categories or physical activity levels, excluding deaths within the first three years.** | | | | | | | | | | | |  |  |
| --- | --- | --- | --- | --- | --- | --- | --- | --- | --- | --- | --- | --- | --- |
|  | | **N** | **Events (n)** | **All-cause mortality**  **HR (95% CI)** | **N** | | **Events (n)** | **CVD mortality**  **HR (95% CI)** | |  |  |  |  |
| **FLI Categories** | |  |  | **Full adjusted Model** |  |  | | **Full adjusted Model** | | |  |  |  |
| < 30 | | 72,553 | 5,858 | 1.00 (Reference) | 75,768 | 1,064 | | 1.00 (Reference) | | |  |  |  |
| 30 - 59 | | 40,950 | 3,166 | **1.13 (1.07─1.19)** | 36,590 | 374 | | **1.21 (1.07─1.37)** | | |  |  |  |
| ≥ 60 | | 23,832 | 1,577 | **1.32 (1.23─1.41)** | 24,977 | 233 | | **1.26 (1.05─1.52)** | | |  |  |  |
| *p* for trend | |  |  | **< 0.001** |  |  | | **< 0.001** | | |  |  |  |
|  |  |  |  |  |  | | |  |  | | | | |
| **MVPA level** | |  |  | **Full adjusted Model** |  |  | | **Full adjusted Model** | | |  |  |  |
| <500 MET-min/week | | 75,768 | 6,376 | 1.00 (Reference) | 72,533 | 944 | | 1.00 (Reference) | | |  |  |  |
| 500 - 999 MET-min/week | | 36,590 | 2,604 | **0.87 (0.83─0.91)** | 40,950 | 514 | | **0.74 (0.66─0.84)** | | |  |  |  |
| ≥1000 MET-min/week | | 24,977 | 1,621 | **0.73 (0.69─0.77)** | 23,832 | 213 | | **0.64 (0.55─0.73)** | | |  |  |  |
| *p* for trend | |  |  | **< 0.001** |  |  | | **< 0.001** | | |  |  |  |
| Full adjusted model: Adjusted for age, sex, smoking status, alcohol consumption, income level, diabetes, dyslipidemia, baseline SBP categories, antihypertensive medication use, BMI categories, and mutually adjusted for PA level and FLI categories, depending on the main exposure of interest. | | | | | | | | | | | | |  |
