# Supplementary material for: Independent and joint associations of fatty liver index and physical activity with mortality in adults with hypertension: a nationwide cohort study
Source: Hypertens Res. 2026 Apr 7;49(6):1839–52. doi: 10.1038/s41440-026-02600-0 (PMC13236582; doi:10.1038/s41440-026-02600-0)
Supplement: Supplementary file 2 — Supplementary Table 2 [file 41440_2026_2600_MOESM2_ESM.docx]

**Supplementary Table 2. Joint association between PA, FLI, and all-cause and CVD mortality, excluding deaths within the first three years.**

| **All-cause mortality** | **Full adjusted model** |
| --- | --- |
| PA < 500 + FLI 60 | 1.00 (Ref.) |
| PA 500-1000 + FLI 60 | 0.88 (0.79-0.99) |
| PA ≥1000 + FLI 60 | 0.87 (0.76-1.00) |
| PA < 500 + FLI 30-59 | 0.87 (0.80-0.95) |
| PA 500-1000 + FLI 30-59 | 0.77 (0.70-0.85) |
| PA ≥1000 + FLI 30-59 | 0.67 (0.60-0.75) |
| PA < 500 + FLI <30 | 0.80 (0.73-0.85) |
| PA 500-1000 + FLI <30 | 0.68 (0.62-0.75) |
| PA ≥1000 + FLI <30 | 0.54 (0.48-0.59) |
| **CVD mortality** | **Full adjusted model** |
| PA < 500 + FLI 60 | 1.00 (Ref.) |
| PA 500-1000 + FLI 60 | 0.95 (0.69-1.31) |
| PA≥1000 + FLI 60 | 0.87 (0.59-1.27) |
| PA < 500 + FLI 30-59 | 1.05 (0.85-1.30) |
| PA 500-1000 + FLI 30-59 | 0.79 (0.61-1.02) |
| PA ≥1000 + FLI 30-59 | 0.69 (0.52-0.92) |
| PA < 500 + FLI <30 | 0.90 (0.72-1.12) |
| PA 500-1000 + FLI <30 | 0.63 (0.49-0.80) |
| PA ≥1000 + FLI <30 | 0.52 (0.40-0.68) |

Full adjusted model: Adjusted for age, sex, current smoking, alcohol consumption, income level, BMI categories, diabetes, dyslipidemia, baseline SBP categories, and antihypertensive medication use.
